# Changes in Seagrass Species Composition in Northwestern Gulf of Mexico Estuaries: Effects on Associated Seagrass Fauna

**DOI:** 10.1371/journal.pone.0107751

**Published:** 2014-09-17

**Authors:** Brandon R. Ray, Matthew W. Johnson, Kirk Cammarata, Delbert L. Smee

**Affiliations:** Texas A&M – University Corpus Christi Department of Life Sciences, Corpus Christi, Texas, United States of America; Dauphin Island Sea Lab; University of South Alabama, United States of America

## Abstract

The objective of this study was to measure the communities associated with different seagrass species to predict how shifts in seagrass species composition may affect associated fauna. In the northwestern Gulf of Mexico, coverage of the historically dominant shoal grass (*Halodule wrightii*) is decreasing, while coverage of manatee grass (*Syringodium filiforme*) and turtle grass (*Thalassia testudinum*) is increasing. We conducted a survey of fishes, crabs, and shrimp in monospecific beds of shoal, manatee, and turtle grass habitats of South Texas, USA to assess how changes in sea grass species composition would affect associated fauna. We measured seagrass parameters including shoot density, above ground biomass, epiphyte type, and epiphyte abundance to investigate relationships between faunal abundance and these seagrass parameters. We observed significant differences in communities among three seagrass species, even though these organisms are highly motile and could easily travel among the different seagrasses. Results showed species specific relationships among several different characteristics of the seagrass community and individual species abundance. More work is needed to discern the drivers of the complex relationships between individual seagrass species and their associated fauna.

## Introduction

Coastal areas worldwide are experiencing loss of seagrass habitat, and this loss has been linked to human activities such as excessive nutrient inputs and overfishing [1]–[4]. Besides loss of seagrass habitat, seagrass species composition is changing in some areas, which may adversely affect faunal assemblages [5], [6]. For example, in North Carolina USA, eel grass (*Zostera marina*) is being displaced by shoal grass (*Halodule wrightii*), and faunal diversity and abundance is significantly less in meadows dominated by shoal grass [5]. Changes in the type of seagrasses inhabiting an area can result from changes in environmental conditions caused by climate change by other anthropogenic activities such as nutrient loading or dredging [7], but also can occur naturally by succession [8]. Seagrasses serve as nursery habitats, predation refuges, food resources, and surface for epiphyte attachment [9]–[12]. They are also important to ecosystem dynamics by providing primary production, stabilizing sediments, and regulating hydrodynamic forces [13]–[16]. Changes in seagrass species composition as well as loss of seagrass habitat may result in a loss of these important functions.

Shoal grass is the most abundant seagrass species in the northern Gulf of Mexico and is a preferred seagrass habitat for fish [6] as well as a food source for red headed ducks (*Athya americana*) [9]. Shoal grass coverage is declining as other seagrass species are becoming more abundant [17], [18]. Shoal grass is a pioneer species that has greater tolerances for low light availability and higher variability in salinity and temperature than other seagrasses [19]–[21]. However, shoal grass is overtaken and shaded by manatee grass (*Syringodium filiforme*) and turtle grass (*Thalassia testudinum*) and is readily displaced by these species when conditions permit their establishment [8], [22]. Shoal grass is denser than the other seagrass species, but, manatee grass is taller, and turtle grass produces wider blades and provides a large surface for epiphyte attachment. Physical differences in seagrass species such as shoot density or leaf morphology can influence predation, secondary production, and species richness and abundance [10], [23]–[26]. Thus, a change from one seagrass species to another can have significant effects on estuarine biodiversity [5], [13], [23].

Over the past several decades, estuaries in the Northwestern Gulf of Mexico have undergone changes in composition of seagrass habitat. Historically, shoal grass, a sub-tropical species, dominated many of these estuaries, but shoal grass is gradually being displaced by other seagrass species. For example, the Laguna Madre located in southern Texas, USA, is one of the largest lagoons in the world and contains more than 600 km^2^ of seagrass habitat [17], [18], [27]. The southern portion of the Laguna Madre has undergone extensive changes, in which shoal grass coverage decreased by ∼60% while turtle grass (*Thalassia testudinum*) and manatee grass increased by a total of ∼50% from 1965 to 1988 [17], [18].

With increasing coastal development, anthropogenic effects on estuarine systems are likely to increase [5], [13]. Changes in historical seagrass compositions, such as those seen in North Carolina and Texas, are likely to occur in other estuaries. To understand the effects of these changes in the western Gulf of Mexico, we measured diversity, abundance, and secondary production of organisms living within monospecific seagrass beds and quantified seagrass characteristics such as shoot density, biomass, and epiphytes to identify relationships among seagrass characteristics and faunal composition.

## Materials and Methods

### Study Area and Sites

The study was performed in Redfish Bay State Scientific Area near Aransas Pass, TX, USA during the fall of 2011. Redfish Bay is part of the Mission-Aransas National Estuarine Research Reserve, and is known for its extensive seagrass habitat [18]. The meadows of Redfish Bay are representative of other seagrass meadows in the Gulf of Mexico such as Big Lagoon in Florida, USA [28]. Redfish Bay is separated from the Gulf of Mexico by barrier islands, and exchanges water primarily through the nearby Corpus Christi Ship Channel. The tidal range is small (∼0.5 m), and tides are primarily wind driven. Redfish Bay contains extensive monospecific stands of shoal grass, manatee grass, and turtle grass that are found at similar depths (∼0.5 m). Because these seagrass beds are in close proximity to one another, each experiences similar biotic and abiotic conditions. Redfish Bay is a public area, and no specific permission was required to use this area for scientific research. No endangered or protected species were involved in the study. Collections of fish and invertebrates were approved by the Texas A&M – Corpus Christi Institutional Animal Care and Use Committee (IACUC #07-07). A Texas Parks and Wildlife Collection Permit (SPR-0409-080) allowed organisms to be collected for this study.

Sites were selected that contained monospecific beds of each seagrass species. Seagrass patches were actively selected to be of similar depth and at least 10 m^2^ in area (Table 1). Further, we sought study sites within Redfish Bay where monotypic seagrass beds of different seagrass species could be found within 10 m of one another, permitting use of a block sampling design. Point measurements of temperature, salinity, and dissolved oxygen were made once at each site on three sampling dates using a Hydrolab data Sonde to verify that abiotic conditions between sites were similar (Table 2).

**Table 1:** Mean depth over sampling period in each seagrass patch at each site.

Site	Seagrass Type	Mean Depth (cm)
Site 1	**Turtle Grass**	42.2
	**Manatee Grass**	43.6
	**Shoal Grass**	35.7
Site 2	**Turtle Grass**	35.1
	**Manatee Grass**	39.6
	**Shoal Grass**	28.3
Site 3	**Turtle Grass**	45.0
	**Manatee Grass**	36.4
	**Shoal Grass**	33.4
Site 4	**Turtle Grass**	34.3
	**Manatee Grass**	31.9
	**Shoal Grass**	26.1
Site 5	**Turtle Grass**	36.2
	**Manatee Grass**	38.8
	**Shoal Grass**	26.1

**Table 2:** Point estimates of temperature, salinity, and dissolved oxygen at each study location by date in Redfish Bay, TX, USA in fall of 2011.

Study location		1	2	3	4	5
**Temperature (°C)**	**10/3/2011**	24.6	26.66	27.53	27.13	27.57
	**10/15/2011**	25.26	26.16	25.85	26.47	26.66
	**11/5/2011**	17.21	17.14	17.96	19.85	20.04
**Salinity (PPT)**	**10/3/2011**	39.56	38.66	38.69	39.97	39.87
	**10/15/2011**	36.22	37.65	38.04	39.12	39.27
	**11/5/2011**	38.25	39.37	38.39	37.93	37.74
**Dissolved Oxygen (ml/l)**	**10/3/2011**	6.27	7.11	7.46	8.65	9.91
	**10/15/2011**	5.96	7.15	6.38	6.3	6.28
	**11/5/2011**	7.07	6.49	6.97	8.78	6.75

### Seagrass Habitat Characterization

The aboveground (AG) biomass and shoot density were measured in each seagrass patch using a core sampler. Three cores, 13.2 cm in diameter were haphazardly taken from each patch on 11/05/2011. Cores were taken deep enough as to include the entire below ground portion (∼15 cm). In the lab, each seagrass leaf was separated and rinsed to remove sediment. First, the total number of shoots in each core was counted. Then, five whole shoots were haphazardly selected to be used for estimation of above ground (AG) biomass. The selected shoots were cut with a razor blade to separate the AG and the below ground (BG) portion of each shoot. The AG portions were then wrapped in individual pre-weighed aluminum foil packets and dried to a constant weight at 70°C. Once dried, AG portions were weighed to the nearest 0.0001 g. AG biomass (total weight of 5 shoots) and shoot density per core were then analyzed by 1-way ANOVA with seagrass species as a fixed factor.

### Epiphyte Characterization

Epiphyte abundance in each patch was estimated by fluorescence emission [29]. For each sample date, three shoots were haphazardly selected from each patch. The shoots from each patch were placed in separate plastic containers and stored in a cool dark place until processing. The epiphytes from the AG portion of the shoot were carefully scraped off the blade surface using a glass slide and a razor blade. Care was taken to not scrape off plant material with the epiphytes. The scraped epiphytes were recovered in a final volume of 25 ml of deionized (DI) water and a 0.3 ml subsample was removed and stored dark at 4°C for three days. Aliquots (0.05 ml) were pipetted into an equal volume of DI water in the wells of a 96 well optical plate. Two fluorescence emission scans were then obtained with a Typhoon 9410 fluorescence scanner (GE Healthcare), one scan with 633 nm red excitation (red) and the other with 532 nm green excitation (green). Red-excited fluorescence detects all photosynthetic organisms, especially green algae and seagrass plant material, if present, because of the presence of chlorophyll. Green-excited fluorescence detects primarily red algae, but also diatoms and some cyanobacteria because of the presence of phycoerythrin and fucoxanthin accessory pigments. The remaining scraped seagrass blades and epiphyte material were then dried for one week at 70°C and weighed to 0.0001 mg. Finally, the epiphyte DW biomass for each epiphyte sample was corrected by adding 1.2% of the measured epiphyte DW biomass to compensate for the 0.3 ml sub sample removed from the total 25 ml volume.

Analysis of the epiphyte scans was performed with ImageQuant software version 5.2. Each fluorescence scan was analyzed for pixel volume (fluorescence intensity) detected by the red-excited and green-excited scans. Pixel volumes were then analyzed by 2-way block ANOVA as the ratio of red-excited scan volume to green-excited scan volume. To compensate for surface area differences per shoot among turtle grass, manatee grass, and shoal grass shoots, epiphyte volume of turtle grass and manatee grass were normalized to the average surface area of a shoal grass shoot (247 mm^2^). To normalize to surface area, the average blade width was obtained from prior research [30], and length was obtained from seagrass cores collected in the study. The average blade dimensions were then used to calculate the average surface area of shoal grass, manatee grass, and turtle grass shoots. Shoal grass and turtle grass have relatively rectangular blade morphologies, while manatee grass blades are cylindrically shaped. Thus, shoal grass and turtle grass surface area was calculated as the area of a rectangle multiplied by two, and manatee grass leaf surface area was estimated using the formula for the surface area of a cylinder. Finally, epiphyte biomass was estimated for each seagrass species. Epiphyte biomass per core was calculated using epiphyte DW biomass and shoot density estimates from seagrass cores. Epiphyte biomass was then analyzed using a blocked 2-way ANOVA with seagrass species and date as fixed factors in the ANOVA model and site as the blocking factor.

### Seagrass Fauna

Monotypic patches of shoal grass, manatee grass, and turtle grass were sampled on 4 dates in 2011 from September-November (9/12, 10/3, 10/15, and 11/5). This sampling time was selected to coincide with peak recruitment of juvenile fishes, particularly those in the family Sciaenidae due to their importance to the local recreational fishery [31]. On each sampling date, 5 sites in Redfish Bay, each containing a monospecific patch of shoal, manatee, or turtle grass, was sampled so that each species of seagrass was sampled 5 times on 4 different dates.

Fishes and other nekton were collected using an epibenthic sled (mesh 500 microns) pulled by hand to sample 10 m^2^ of seagrass habitat [32]–[34]. Collections with the epibenthic sled were taken haphazardly within each patch. All fauna were removed from the epibenthic sled, placed in ethanol, and returned to Texas A&M University-Corpus Christi for sorting, identification, and enumeration. Organisms were identified to lowest possible taxon.

Faunal abundance and diversity were analyzed using both multivariate and univariate analyses. Multivariate analysis was performed using PRIMER software. First, a Bray-Curtis Similarity Matrix was constructed. To examine community composition of macrofauna among seagrass species, a 2-way nested analysis of similarity (ANOSIM) with the factors site, seagrass type, and date was performed. The factor seagrass was nested within site. Multi-dimensional scaling (MDS) was also performed to compare community composition of macrofauna among seagrass species. Both ANOSIM and MDS are useful analyses for investigation of differences in community composition [35]. We also performed a SIMPER analysis in PRIMER to determine which faunal species contributed to the similarity and dissimilarity between seagrass beds of differing species [35].

The abundance and diversity of fauna were compared among seagrass species using a blocked 2-way ANOVA with seagrass species and date as fixed factors in the ANOVA model and site as the blocking factor [36]. Statistical analyses were performed using SAS version 9.2. Raw data were log(x+1) transformed to meet assumptions of normality. For both total faunal abundance and biodiversity, one number was calculated for each patch on each date, and then 2-way blocked ANOVA was performed. Biodiversity was calculated using Shannon's diversity index (H′). Separate ANOVAs using the same factors were also performed to compare abundances of fish, shrimp, and crabs among different seagrass species. Lastly, individual species of economic importance such as blue crabs (*Callinectes sapidus*), Penaeidae (penaeid shrimp <40 mm), brown shrimp (*Farfantepenaeus aztecus*), and red drum (*Sciaenops ocellatus*), and individual species comprising more than 5% of the total collection were analyzed in this manner. If a significant interaction occurred between seagrass species and date, a simple main effects 1-way ANOVA was performed [37]. Tukey's multiple comparisons test of the means was performed on significant p-values≤0.05.

### Secondary Production

Secondary production was estimated for all seagrass dependent species of fish, shrimp, and crabs collected using the formula P = log_10_^−1^(0.66–0.726log_10_L+log_10_B) where P = annual production (g AFDW m^−2^ yr^−1^), B = biomass (g AFDW m^−2^), and L = lifespan in years [38]. First, dry weight (DW) was obtained by drying all specimens to a constant weight at 80°C to the nearest 0.0001 g. Then, ash weight (AW) was obtained for all organisms, except shrimp, by ashing the dried organisms in a muffle furnace at 500°C for 5 hours and weighing to the nearest 0.0001 g. All samples were placed in desiccators to cool before being weighed for DW and AW. For fish and crabs the AFDW was then calculated by subtracting AW from DW (AFDW = DW-AW). In shrimp, the AFDW was estimated as DW x 0.9 because of the low amount of inorganic matter in shrimp [36].

Analysis of secondary production data was performed using the same blocked 2-way model that was used in analysis of biodiversity and abundance. Estimates of total secondary production, secondary production of fish, secondary production of shrimp, secondary production of crabs, and secondary production of commercially and/or recreationally important species was performed using a blocked 2-way ANOVA. Tukey's multiple comparisons test of the means was then performed on all significant p-values. Analysis was performed on a range of the minimum and maximum lifespans published on time spent in seagrasses in years for fish, shrimp, and crabs developed by [38], [39]. In this equation, lifespan is a variable, but lifespans are difficult to ascertain for invertebrates. We therefore used minimum and maximum lifespans published for each species to provide a range of secondary production estimates.

### Relationships among Seagrass Species Attributes, Epiphyte Differences, and Fauna

To investigate dependency of total fish, shrimp, and crab abundance on habitat characteristics, total epiphyte load (pixel volume), AG biomass, and shoot density, forward stepwise regression was performed using SigmaPlot version 11.0 (Systat Software Inc., San Jose California USA). Forward stepwise regression was performed for total fish, shrimp, and crab abundance data, as well as groups and species that had significant differences in abundance among seagrasses.

## Results

### Seagrass Habitat Characterization

**Figure 1:**
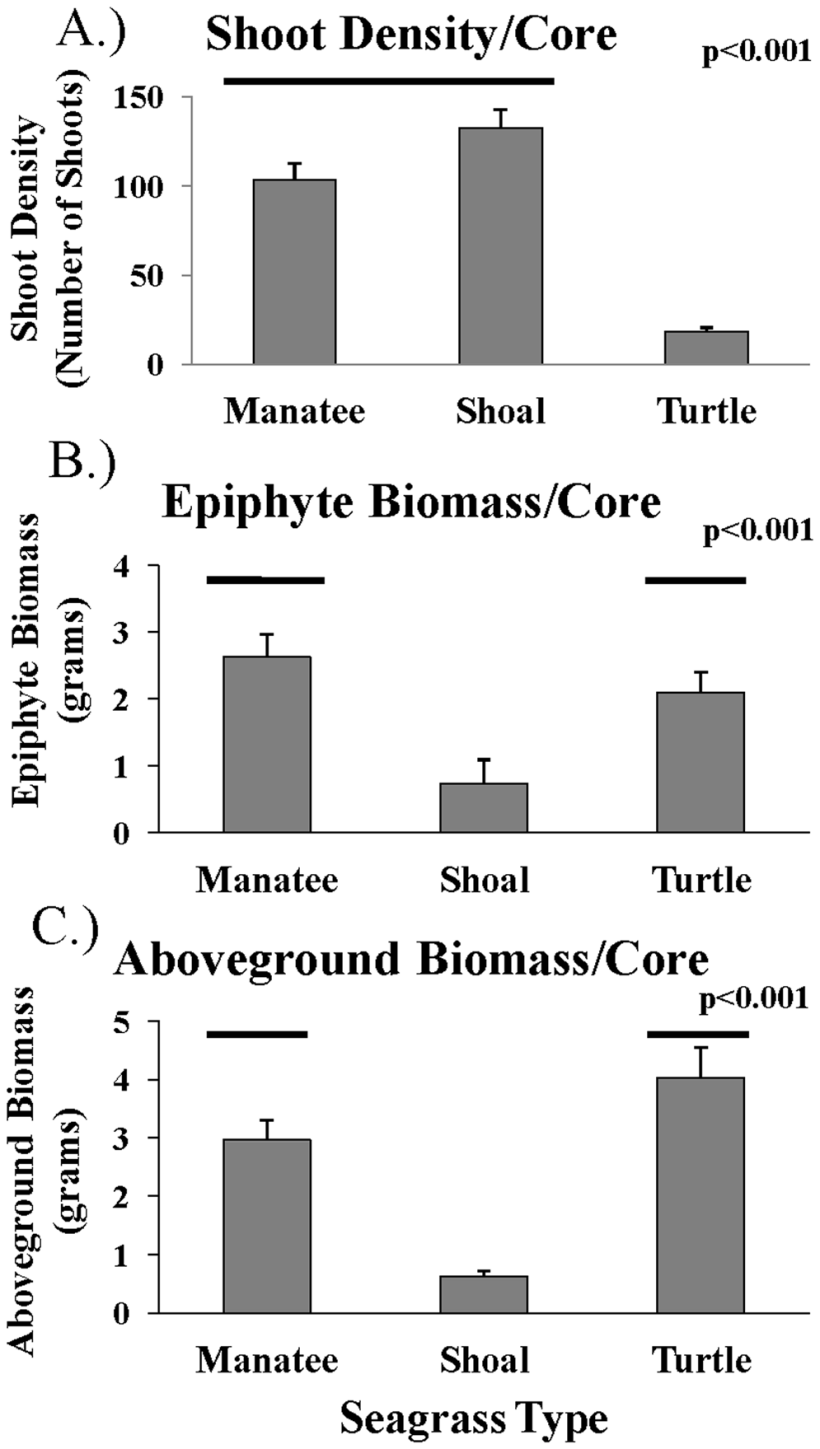
Mean (+ SE) A) shoot density B) epiphyte biomass per core and C) aboveground biomass per core among 3 seagrass species.

Seagrass AG biomass (F_2,42_ = 23.9, p<0.001) and shoot density (F_2,42_ = 59.8, p<0.001) were different among seagrass species. Tukey's HSD test indicated that shoal grass had significantly lower AG biomass than manatee grass and turtle grass. In contrast, turtle grass had significantly fewer shoots per core than manatee grass and shoal grass (Figure 1).

### Epiphyte Characterization

**Figure 2:**
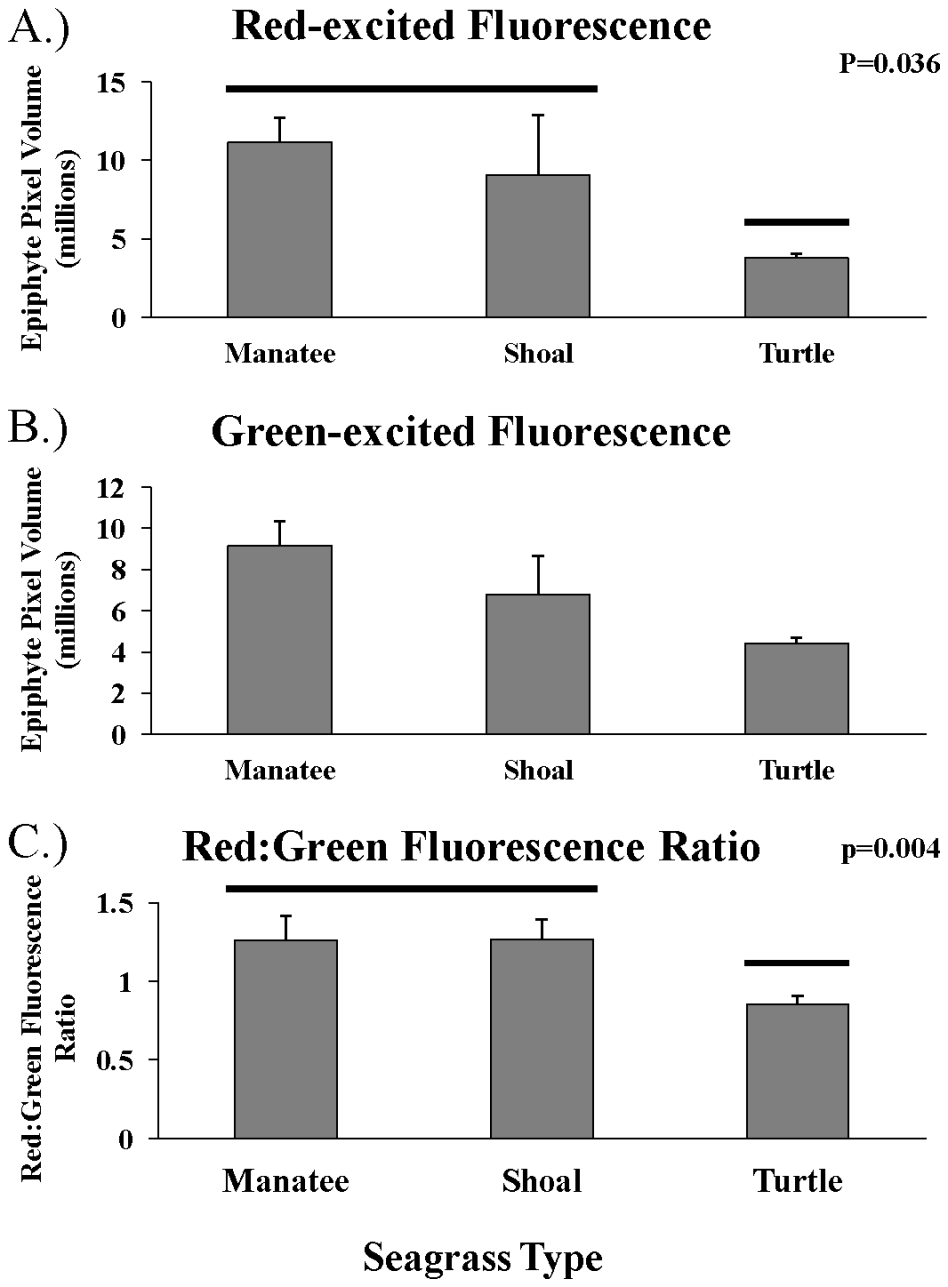
Mean epiphyte pixel volume (+SE) standarzied to seagrass leaf surface area (247 mm^2^), detected by flourometry among 3 seagrass species. A) red-excited fluorescence B) green-excited fluorescence and C) ratio of red to green fluorescence. Red-excited fluorescence detects all photosynthetic organisms, green-excited fluorescence detects primarily red algae but also diatoms and some cyanobacteria.

Manatee grass had significantly more epiphytes per blade surface area than turtle grass, but not shoal grass, for epiphytes detected by red-excited fluorescence (F_2,2_ = 70.2, p<0.001, Figure 2, Table 3). Comparison of the ratio of red-excited fluorescence to green-excited fluorescence revealed that there were significant differences among seagrass species (F_14,59_ = 6.39, p = 0.004, Table 3). Tukey's test showed that manatee grass and shoal grass had ratios that were significantly higher than those of turtle grass (Figure 2). All ratios were relatively low (<1.4), indicating that red algae appear to be dominant. The significantly higher ratios of red-excited fluorescence to green-excited fluorescence on shoal and manatee grass indicate that there may be differences in epiphytic communities among seagrass species. Specifically, there may be more green algae, brown algae, diatoms, and/or slightly less red algae on manatee and shoal grass blades than those of turtle grass. Tukey's test indicated that shoal grass had significantly less epiphyte biomass per core than manatee grass and turtle grass (F_2,37_ = 14.36, p<0.001; Figure 1, Table 3).

**Table 3:** 2-way blocked analysis of variance (ANOVA) for epiphytes including red florescence, ratio of red∶green epiphyte florescence, and epiphyte biomass.

			Seagrass		Date		Seagrass*Date	
Category	DF	Error	F	P	F	P	F	P
**Red florescence**	15	44	70.2	**<0.0001**	1.28	0.29	2.02	0.08
**Red∶green florescence ratio**	15	44	6.39	**0.004**	2.65	0.06	1.47	0.21
**Epiphyte weight**	12	27	13.8	**<0.0001**	4.06	**0.03**	1.17	0.34

### Seagrass Fauna

**Figure 3:**
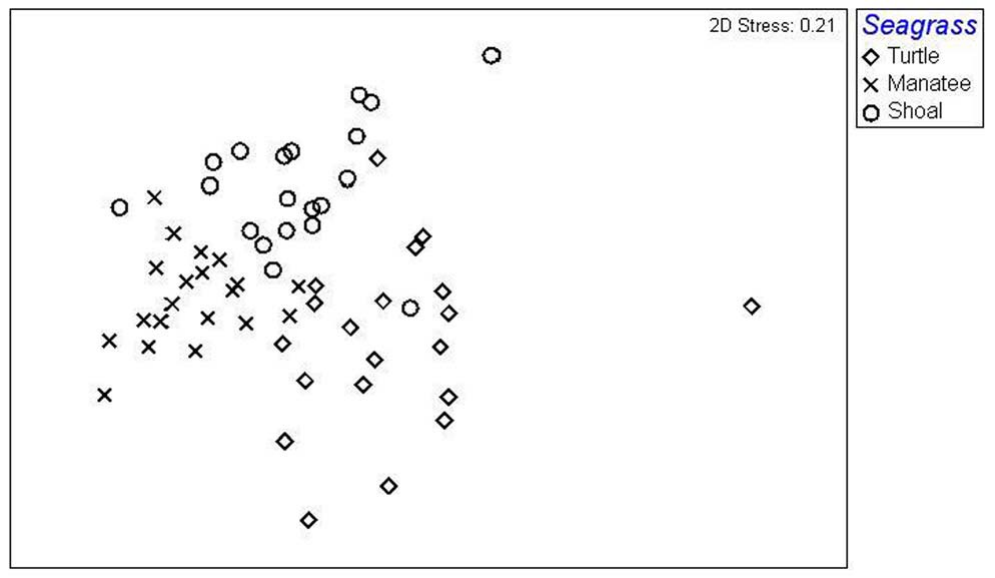
MDS ordination of fish, shrimp, and crabs among 3 seagrass species.

Two-dimensional MDS indicated clustering of samples among seagrass species with a stress level of 0.21 (Figure 3). Significant differences in assemblages of fish, shrimp, and crabs among the different seagrass species were found using ANOSIM (Global R = 0.646, P<0.001). Pairwise comparisons among the seagrass species indicated differences in community composition between turtle grass and manatee grass (Global R = 0.748, P = 0.008), turtle grass and shoal grass (Global R = 0.604, P = 0.008), and manatee grass and shoal grass (Global R = 0.712, P = 0.008). Global R values of 1 indicate that similar samples were found within the same seagrass species while R values of 0 indicate that similar samples were found within different seagrass species. Our values were above 0.6, which indicates that most of our similar samples were found within the same seagrass species, providing evidence that the faunal composition was not similar between different seagrass species. SIMPER analysis was used to further quantify similarity between groups and revealed more than 50% faunal dissimilarity between seagrass species (Table 4). Shoal grass was 51% dissimilar from turtle grass and 59% dissimilar from manatee grass. Manatee and shoal grass were 56% dissimilar. Dissimilarities were primarily driven by abundances of grass shrimp, Penaeid shrimp, arrow shrimp, gobies, and crabs in the family Xanthidae (Table 4).

**Table 4:** Results from SIMPER analysis.

	Shoal Grass		Manatee Grass		Turtle Grass		Shoal and Manatee Grass	Shoal and Turtle Grass	Manatee and Turtle Grass
	Mean Density	% Similarity	Mean Density	% Similarity	Mean Density	% Similarity	% Dissimilarity	% Dissimilarity	% Dissimilarity
Palaemonetes (Grass Shrimp)	207.6	33.84	244.05	31.8	197.4	48.42	25.94	32.19	22.44
*Farfantepenaeus aztecus* (Brown Shrimp)	—	—	—	—	8.75	1.92	—	1.63	—
Penaeidae	33.1	8.39	—	—	10.95	1.7	3.63	5.94	2.48
*Tozeuma carolinense* (Arrow Shrimp)	—	—	191.3	20.31	—	—	23.44	1.73	24.35
*Gobionellus boleosoma* (Darter goby)	8.2	2.1	—	—	—	—	—	1.83	—
Xanthidae	—	—	—	—	—	—	—	1.94	1.46
*Gobiosoma robustum* (Goby)	—	—	—	—	—	—	—	1.73	—

**Figure 4:**
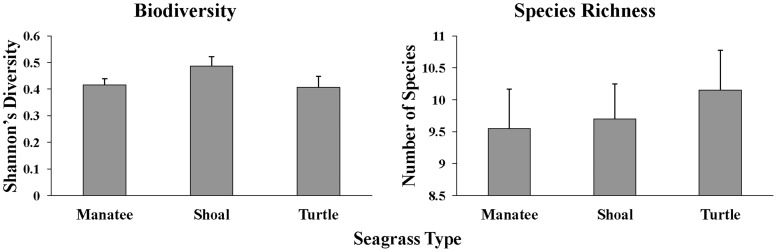
Mean (+SE) Shannon's Diversity Index (H′) and species richness among 3 seagrass species.

**Figure 5:**
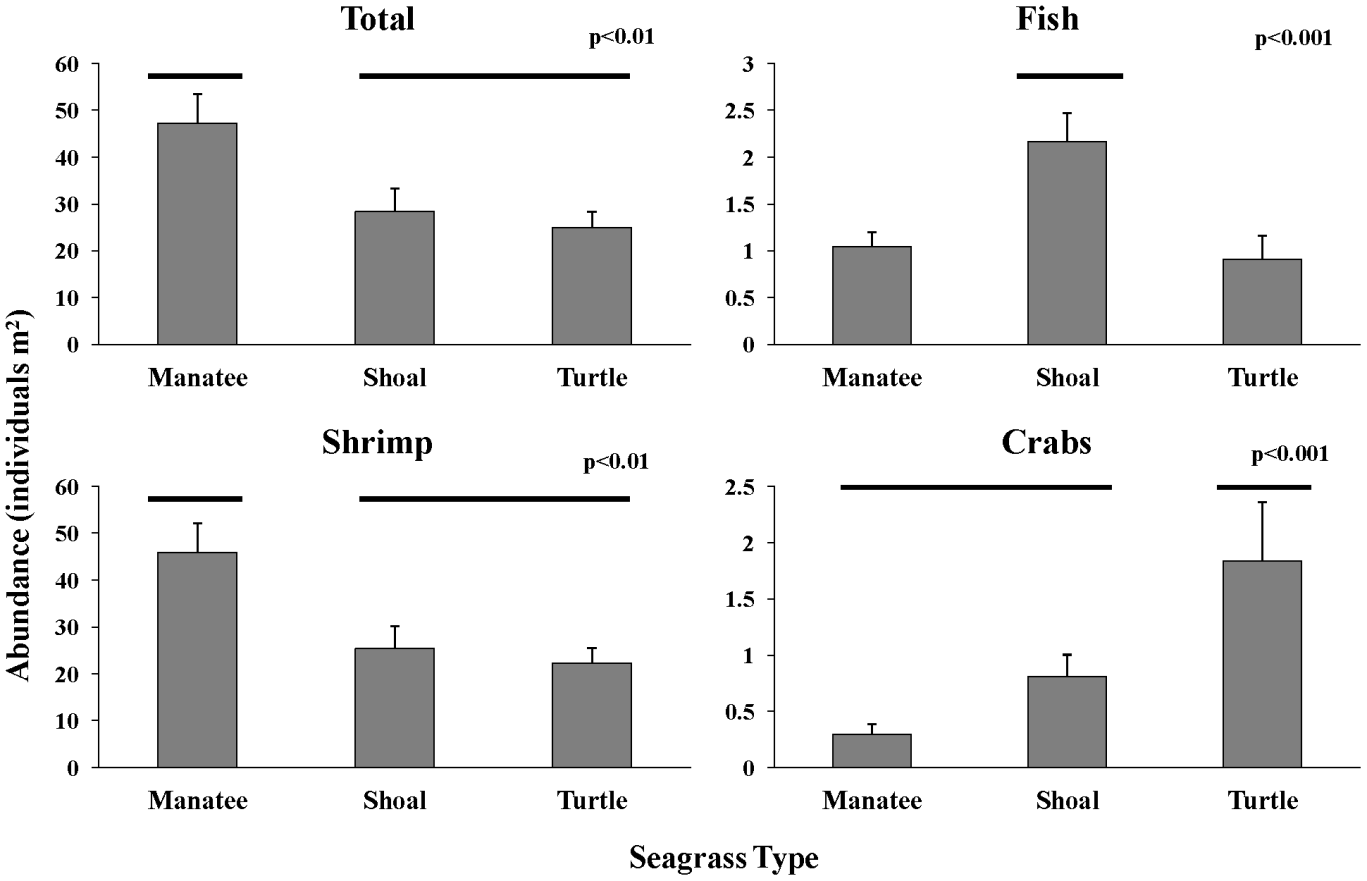
Mean (+SE) abundance of organisms collected among 3 seagrass species.

Biodiversity of macrofauna as measured with the Shannon diversity index was not significantly different among seagrass species (F_15,59_ = 1.96, p = 0.15, Figure 4) nor was species richness (F_15,59_ = 1.05, p = 0.25, Figure 4). Total faunal density and density of fish, crabs, and shrimp were significantly different among seagrass species (Figure 5), as were the abundances of gobies (Gobiidae), pipefish and seahorses (Sygnathidae), Penaeidae (penaeid shrimp<40 mm), brown shrimp (*Farfantepenaeus aztecus*), arrow shrimp (*Tozeuma carolinense*), mud crabs (Xanthidae), blue crabs (*Callinectes sapidus*), and *Callinectes* spp. (Table 5).

**Table 5:** 2-way blocked analysis of variance (ANOVA) of diversity and abundance (Significance at α≤0.05).

			Seagrass		Date		Seagrass*Date	
Category	DF	Error	F	P	F	P	F	P
**Biodiversity (H′)**	59	44	1.96	0.15	4.10	**0.01**	0.64	0.6943
**Total Abundance**	59	44	6.05	**<0.01**	0.74	0.54	0.38	0.8876
**Species Richness**	59	44	0.25	0.78	0.42	0.74	0.69	0.6577
**Fish Abundance**	59	44	8.87	**<0.001**	1.16	0.34	1.09	0.3853
**Shrimp Abundance**	59	44	7.01	**<0.01**	0.66	0.58	0.39	0.8824
**Crab Abundance**	59	44	13.93	**<.001**	2.19	0.10	2.44	**0.04**
**Gobiidae**	59	44	8.98	**<0.001**	1.13	0.35	0.45	0.84
**Syngnathidae**	59	44	5.16	**<0.01**	3.33	**0.03**	2.00	0.09
***Sciaenops ocellatus***	59	44	0.93	0.40	11.10	**<0.001**	0.51	0.80
***Farfantepenaeus aztecus***	59	44	9.10	**<0.001**	5.82	**0.002**	1.85	0.11
**Penaeidae (<40 mm)**	59	44	15.16	**<0.001**	11.73	**<0.001**	0.86	0.53
***Palaemonetes*** ** sp.**	59	44	0.72	0.49	1.33	0.28	0.50	0.80
***Tozeuma carolinense***	59	44	107.86	**<0.001**	1.86	0.15	0.42	0.86
**Xanthidae**	59	44	16.30	**<0.001**	1.86	0.15	2.05	0.08
***Callinectes sapidus***	59	44	11.96	**<0.001**	0.86	0.4712	0.72	0.6379
***Callinectes*** ** sp.**	59	44	7.09	**<0.01**	4.00	**0.01**	1.51	0.1979

**Figure 6:**
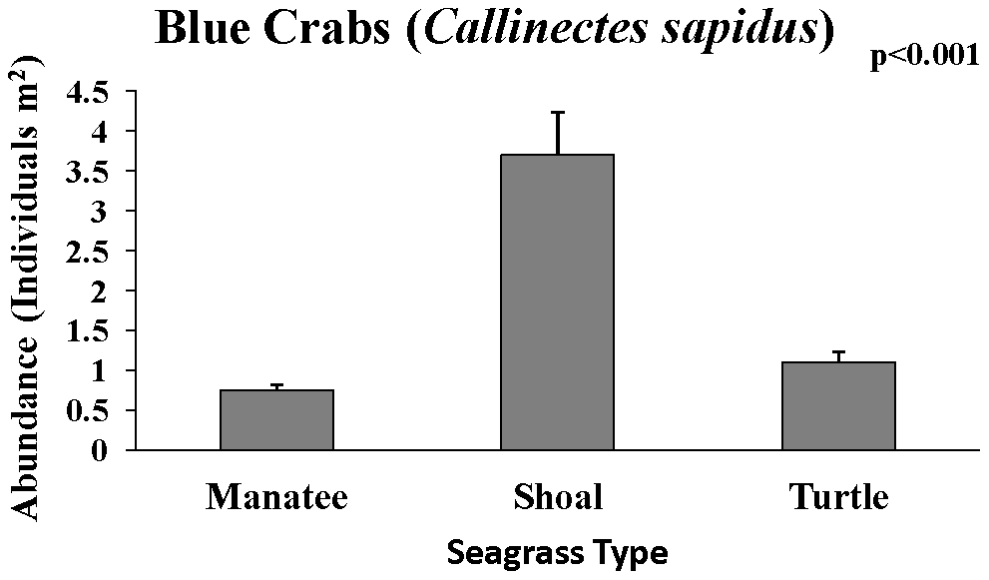
Mean (+SE) of blue crabs collected in each seagrass species.

**Figure 7:**
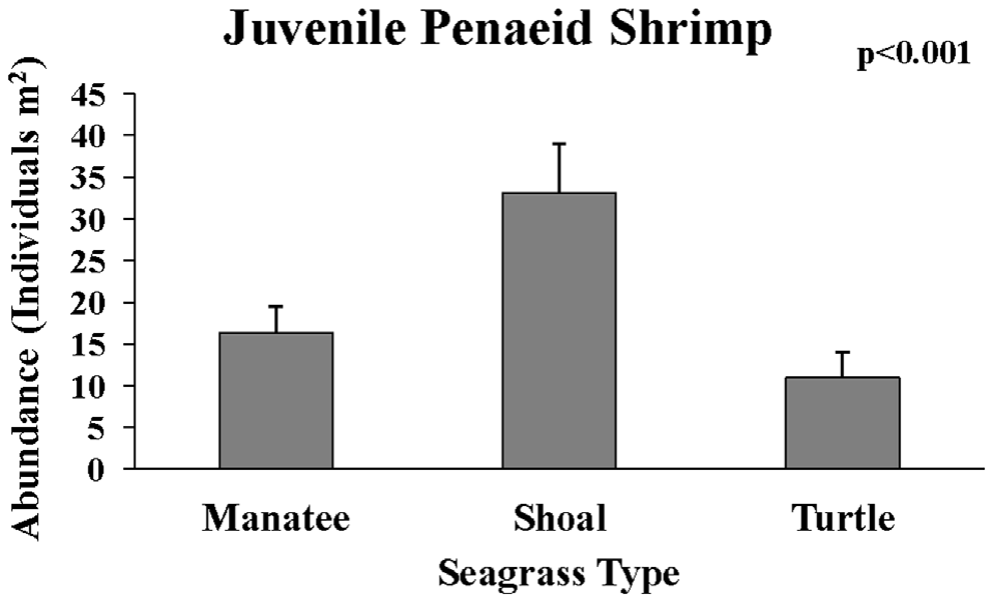
Mean (+SE) of shrimp in family Penaeidae collected in each seagrass species.

Total density of all combined species was highest in manatee grass (F_15,59_ = 6.05, p<0.01), and significant pairwise differences were detected with Tukey's HSD test. Shrimp had the highest density of any group and were most abundant in manatee grass (F_15,59_ = 7.01, p<0.01), which accounted for the overall highest density of organisms in manatee grass. Fish had highest density in shoal grass (F_15,59_ = 8.87, p<0.001), and crabs had the highest density in turtle grass (F_15,59_ = 13.93, p<0.001). Three groups: blue crabs (F_15,59_ = 12.41, p<.001, Figure 6), gobies (F_15,59_ = 8.98, p<0.001), and Penaeidae (F_15,59_ = 15.16, p<.001, Figure 7) had highest density in shoal grass. Arrow shrimp had significantly higher density in manatee grass (F_15,59_ = 107.86, p<.001), and mud crabs had significantly higher density in turtle grass (F_15,59_ = 16.30, p<0.001). Pipefish and seahorses had lowest density in turtle grass but their density was not statistically different between manatee and shoal grass (F_15,59_ = 5.16, p<0.01). Brown shrimp had significantly lower density in shoal grass than in the other seagrass habitats (F_15,59_ = 9.10, p<0.001). Of the groups investigated, only grass shrimp (*Palaemonetes sp.*, F_15,59_ = 0.72, p = 0.49) and red drum (*S. ocellatus*, F_15,59_ = 0.93, p = 0.40) did not show significant differences among seagrass species.

Date was a significant factor for biodiversity and the density of red drum, sygnathids, brown shrimp, and penaeid shrimp (Table 5). Tukey's HSD test indicated that biodiversity (F_15,59_ = 4.10, p<0.05) and density of red drum (F_15,59_ = 11.10, p<.001) were higher on the last sampling date than the other three sampling dates. Density of pipefish and seahorses were higher on the first sampling date than the last sampling date (F_15,59_ = 3.33, p<0.05). Density of brown shrimp was lower on the last sampling date compared to the other three sampling dates (F_15,59_ = 5.82, p<0.01). Lastly, density of penaeid shrimp was higher on the first sampling date than the last two sampling dates (F_15,59_ = 11.73, p<.001).

One significant interaction occurred between seagrass habitat type and date for crab density (F_15,59_ = 2.44, p = 0.04, Table 5). Further analysis by simple main effects revealed that crab density was significantly higher in turtle grass than the other seagrass species (F_3,59_ = 9.31, p<0.001), but was not different among the four sample dates (F_3,59_ = 1.15, p = 0.34).

### Secondary Production

Total secondary production was significantly higher in manatee grass for both minimum (F_15,59_ = 8.66, p<0.001) and maximum (F_15,59_ = 9.13, p<0.001) lifespan estimates. Among the major groups, shrimp were the only group with significant differences in secondary production among seagrass species. ANOVA results for minimum and maximum secondary production estimates gave similar results (Tables 6, 7). Secondary production of shrimp was highest in manatee grass (F_15,47_ = 17.24, p<.001). Two shrimp categories, arrow shrimp and grass shrimp, had significant differences in secondary production estimates among seagrass species. Secondary production of arrow shrimp, like that of total and shrimp secondary production was also highest in manatee grass (F_14,43_ = 11.60, p<0.001). Grass shrimp, had significantly higher secondary production in manatee grass than in turtle grass but not in shoal grass (F_15,57_ = 3.73, p = 0.03).

**Table 6:** 2-way blocked analysis of variance (ANOVA) of secondary production estimates for minimum lifespan estimates in years (Fish = 0.5, Shrimp = 0.17, Crabs = 1, Significance at α≤0.05).

			Seagrass		Date		Seagrass*Date	
Category	DF	Error	F	P	F	P	F	P
**Total**	15	44	8.66	**<0.001**	0.85	0.47	0.58	0.74
**Fish**	15	42	1.09	0.35	0.66	0.58	1.28	0.29
**Shrimp**	15	42	17.24	**<0.001**	0.66	0.58	0.40	0.88
**Crabs**	15	35	1.96	0.16	0.29	0.83	2.69	**0.03**
**Penaeidae**	15	38	1.77	0.18	7.11	**0.001**	0.27	0.95
**Palaemonetes**	15	42	3.73	**0.03**	0.04	0.99	0.42	0.86
***Tozeuma carolinense***	14	29	11.60	**<0.001**	0.02	0.99	0.04	0.99

**Table 7:** 2-way blocked analysis of variance (ANOVA) of secondary production estimates for maximum lifespan estimates in years (Fish = 1.5, Shrimp = 0.42, Crabs = 3, Significant at α≤0.05).

			Seagrass		Date		Seagrass*Date	
Category	DF	Error	F	P	F	P	F	P
**Total**	15	44	9.13	**<0.001**	0.85	0.47	0.60	0.73
**Fish**	15	42	1.09	0.35	0.66	0.58	1.28	0.29
**Shrimp**	15	42	17.24	**<.001**	0.66	0.58	0.40	0.88
**Crabs**	15	35	1.96	0.16	0.29	0.83	2.69	**0.03**
**Penaeidae**	15	38	1.77	0.23	7.11	**<0.001**	0.27	0.95
**Palaemonetes**	15	42	3.73	**0.03**	0.04	0.99	0.42	0.86
***Tozeuma carolinense***	14	29	11.60	**<0.001**	0.02	0.98	0.04	0.99

### Relationships among Seagrass Species Attributes, Epiphyte Differences, and Fanua

Forward stepwise regression indicated significant relationships for total density of fish and crabs, as well as, the individual densities of gobies, pipefish and seahorses, blue crabs, mud crabs, arrow shrimp, and shrimp in the family Penaeidae with one or more of the seagrass plant characteristics including epiphyte volume, AG biomass, and shoot density (Table 8). Density of fish (F_1,55_ = 11.10, p = 0.002, R^2^ = 0.17), gobies (F_1,55_ = 9.30, p = 0.004, R^2^ = 0.15), and blue crabs (F_1,55_ = 6.64, p = 0.013, R^2^ = 0.11) showed a significant relationship with total epiphyte volume. Density of all crabs (F_1,55_ = 16.57, p<0.001, R^2^ = 0.23) and density of mud crabs (F_1,55_ = 25.12, p<0.001, R^2^ = 0.31) showed a significant relationship with AG biomass. Density of penaeid shrimp (F_1,55_ = 7.95, p = 0.007, R^2^ = 0.13) and pipefish and seahorses (F_1,55_ = 8.77, p = 0.005, R^2^ = 0.14) showed a significant relationship with average shoot density. Arrow shrimp exhibited a significant relationship with both total epiphyte volume (F_2,54_ = 4.70, p = 0.035, R^2^ = 0.27) and AG biomass (F_2,54_ = 18.78, p<0.001, R^2^ = 0.20). Despite, the significant relationships of density with individual plant characteristics, R^2^ values were low, suggesting that numerous factors are likely influencing the abundance of each species. Differences in seagrass morphological characteristics accounted for a minimal amount of variation in abundance of seagrass fauna. The strongest correlations are total crab density with AG biomass (R^2^ = 0.23), specifically the correlation between mud crab density and AG biomass (R^2^ = 0.31), as well as the correlation between arrow shrimp density and both epiphyte volume (R^2^ = 0.27) and above ground biomass (R^2^ = 0.20).

**Table 8:** Forward stepwise regression between group and species abundances and the plant characteristics epiphyte volume, aboveground biomass, and shoot density.

Plant Characteristic	Category	F	P	R^2^
**Epiphyte Fluorescence Volume**	**Fish Abundance**	11.10	0.002	0.17
	**Gobiidae**	9.30	0.004	0.15
	***Callinectes sapidus***	6.64	0.013	0.11
	***Tozeuma carolinense***	4.70	0.035	0.27
**Aboveground Biomass**	**Crab Abundance**	16.57	<0.001	0.23
	**Xanthidae**	25.12	<0.001	0.31
	***Tozeuma carolinense***	18.78	<0.001	0.20
**Shoot Density**	**Penaeidae**	7.95	0.007	0.13
	**Sygnathidae**	8.77	0.005	0.14

Significant at α≤0.05.

## Discussion

As with many other ecosystems, coastal marine environments are experiencing unprecedented natural and anthropogenic threats, resulting in declining critical habitats and biodiversity along with losses of ecosystem services [3], [5], [13], [37]. To understand consequences of anticipated changes, it is important to establish baseline data in a variety of habitats. Long recognized as important to many coastal processes, sub-tropical seagrasses have experienced community-level changes that may alter their structure and function within the coastal ecosystem [5], [18]. Changes at this level will not only alter plant characteristics, but also community dynamics of the many organisms that rely on seagrass meadows as nursery grounds and the movement of energy through coastal ecosystems [10], [40]. Here, we quantified seagrass-related macrofaunal species and examined their relationships with specific seagrass characteristics in a location experiencing substantial shifts in seagrass species composition as manatee grass expands its range northward. Results suggest that communities inhabiting seagrass beds differed among seagrass species and that as manatee grass out competes shoal grass there may be ecosystem-level changes.

The basis for any ecosystem-level changes within sub-tropical seagrass meadows may be from changes in the morphology of the different grasses and how they facilitate the transfer of energy. In the northwest Gulf of Mexico, increases in manatee grass abundance resulted in a habitat that had characteristics of both turtle grass and shoal grass [17]. Morphologically manatee grass blade characteristics and shoot density are similar to shoal grass, but the AG biomass is more similar to turtle grass. The epiphyte load on manatee grass is also more similar to shoal grass with fewer non-palatable red algae compared to turtle grass. Ultimately, the major ecological changes to this ecosystem may be due to changes in the complexity of the physical structure of seagrass habitats, because physical differences such as shoot density can influence predation, secondary production, and species richness and abundance [10], [23]–[26]. Manatee grass is taller and less dense than shoal grass, and thus community differences may be related to these characteristics and their influence upon epiphyte abundance and trophic interactions. Compared to unvegetated habitats, seagrasses have been shown to increase recruitment, survival, and growth [12], [40], [41], and the greater surface area of manatee grass may favor more epiphytes and greater numbers of grazers [40]. We measured epiphyte loads, AG biomass, and shoot density of turtle, manatee, and shoal grass, because these particular habitat characteristics have been shown to affect abundance of fauna [6], [29], [42]. Yet, we did not find clear patterns between any of these factors and overall community composition, suggesting that many mechanisms specific to different faunal groups are driving observed patterns. Additionally, studies with individual species are necessary to tease apart the mechanisms responsible for the community patterns observed.

Increased abundance of manatee grass will increase the habitat available for settlement of epiphytes. We found a greater proportion of more palatable non-red algae epiphytes on manatee grass, which would provide more food for grazers that consume these epiphytes. We found arrow shrimp to be significantly more abundant and to have higher rates of secondary production in manatee grass, a pattern also noted in Florida [43]. The increased abundance and growth rate of arrow shrimp can be attributed to additional food in the form of epiphytes, greater protection from predators, or a combination of these. Fluorescence indicated that manatee and shoal grass had significantly different algal epiphyte communities than did turtle grass. Furthermore, turtle and manatee grass had greater epiphyte biomass than shoal grass when standardized to leaf area. Turtle and manatee grass may have more food available to mesograzers when compared to shoal grass, but potentially different types of food available from one another as indicated by fluorescence emission differences.

Habitat complexity in seagrasses is considered to be one of the major drivers of community structure for seagrass specific fauna. Research suggests that many motile organisms have the ability to choose preferred microhabitats [44] to increase survival in response to predation pressure [45]. In the Gulf of Mexico, the abundance of grass shrimp and red drum are positively affected by increased seagrass coverage and habitat complexity because either these species prefer more complex habitats, have higher survival in these areas, are more effective foragers, or a combination of these factors [39], [41], [46]–[48]. With greater seagrass biomass comes an increase in available habitat and generally an overall greater faunal abundance [48]. Yet, seagrasses differ in several ways including blade shape, blade surface area, AG biomass, and shoot density as well as physiologically in terms of growth rates, leaf turnover, and nutrient content [49], [50]. The exact benefits each seagrass offers each faunal species appears to be different and complex as differences in seagrass characteristics only accounted for a minimal amount of variation in abundance in our assessment. For example, we found negative relationships between epiphyte load and abundance of fishes and blue crabs. We suspect this is not a causal relationship; but rather these organisms may prefer shoal grass for predator protection or may more readily escape predation pressure as denser habitats can lower predation rates [41].

The seagrass beds sampled in this study were adjacent to one another, and many of the organisms collected are mobile and could easily travel among different monospecific stands of seagrass. Seagrass fauna likely move among seagrass beds as necessary to meet needs including energy acquisition, predator avoidance, and reproduction or mating. Despite the high motility of the sampled species, we noted significant differences in communities inhabiting each seagrass type. We interpret this finding as evidence that individual species prefer certain seagrass species. However, pinpointing the benefits of each seagrass species for each faunal species is challenging and requires significant research far beyond the scope of the study. For example, we found a significant correlation between crab density and AG biomass, but we could not determine if these results were due to greater AG biomass providing better predation refuges of foraging locations for small crabs, enhancing crab reproduction, or facilitating other functions that act synergistically or separately from those listed. Our data clearly show that some organisms like blue crabs and small fishes are more common in shoal grass, and when this grass is replaced by other species the abundance of these organisms may decrease while the abundance of other organisms like arrow shrimp are likely to increase. Long term monitoring will be required for better assessment, and this study provides a baseline for beginning this type of monitoring.
